# The Effect of Maternal Obesity on Breast Milk Fatty Acids and Its Association with Infant Growth and Cognition—The PREOBE Follow-Up

**DOI:** 10.3390/nu11092154

**Published:** 2019-09-09

**Authors:** Andrea de la Garza Puentes, Adrià Martí Alemany, Aida Maribel Chisaguano, Rosa Montes Goyanes, Ana I. Castellote, Franscisco J. Torres-Espínola, Luz García-Valdés, Mireia Escudero-Marín, Maria Teresa Segura, Cristina Campoy, M. Carmen López-Sabater

**Affiliations:** 1 Department of Nutrition, Food Sciences and Gastronomy, Faculty of Pharmacy and Food Sciences, University of Barcelona, 08028 Barcelona, Spain; 2 Institut de Recerca en Nutrició i Seguretat Alimentària UB (INSA-UB), 08921 Barcelona, Spain; 3 Teaching, Research & Innovation Unit, Parc Sanitari Sant Joan de Déu, 08830 Sant Boi, Spain; 4 Nutrition, Faculty of Health Sciences, University of San Francisco de Quito, 170157 Quito, Ecuador; 5 Food Research and Analysis Institute, University of Santiago de Compostela, 15705 Santiago de Compostela, Spain; 6 CIBER Physiopathology of Obesity and Nutrition CIBERobn, Institute of Health Carlos III, 28029 Madrid, Spain; 7 Centre of Excellence for Paediatric Research EURISTIKOS, University of Granada, 18071 Granada, Spain; 8 Department of Paediatrics, University of Granada, 18071 Granada, Spain; 9 CIBER Epidemiology and Public Health CIBEResp, Institute of Health Carlos III, 28029 Madrid, Spain

**Keywords:** maternal obesity, breastfeeding, breast milk, colostrum, mature milk, fatty acids, LC-PUFA, omega-3, omega-6, DHA, AA, children, growth, cognition, early life nutrition, programming

## Abstract

This study analyzed how maternal obesity affected fatty acids (FAs) in breast milk and their association with infant growth and cognition to raise awareness about the programming effect of maternal health and to promote a healthy prenatal weight. Mother–child pairs (*n* = 78) were grouped per maternal pre-pregnancy body mass index (BMI): normal-weight (BMI = 18.5–24.99), overweight (BMI = 25–29.99) and obese (BMI > 30). Colostrum and mature milk FAs were determined. Infant anthropometry at 6, 18 and 36 months of age and cognition at 18 were analyzed. Mature milk exhibited lower arachidonic acid (AA) and docosahexaenoic acid (DHA), among others, than colostrum. Breast milk of non-normal weight mothers presented increased saturated FAs and n6:n3 ratio and decreased α-linolenic acid (ALA), DHA and monounsaturated FAs. Infant BMI-for-age at 6 months of age was inversely associated with colostrum n6 (e.g., AA) and n3 (e.g., DHA) FAs and positively associated with n6:n3 ratio. Depending on the maternal weight, infant cognition was positively influenced by breast milk linoleic acid, n6 PUFAs, ALA, DHA and n3 LC-PUFAs, and negatively affected by n6:n3 ratio. In conclusion, this study shows that maternal pre-pregnancy BMI can influence breast milk FAs and infant growth and cognition, endorsing the importance of a healthy weight in future generations.

## 1. Introduction

In spite of efforts made, as well as existing evidence-based information, for tackling obesity and the burden of the disease, obesity is a societal challenge that is still on the rise, including in women of reproductive age, and this is affecting the health of future generations [1]. Early-life nutrition plays a key role in infant growth and development and has a programming effect related to the appearance of future non-communicable diseases, such as obesity, diabetes and others [2,3]. Breast milk composition and breastfeeding practice are some of the most influential factors of child outcomes [4,5,6]. Even though lactation comprises a relatively short period in the average person’s lifespan, the exposure to breast milk in the first months of life occurs during a very critical period of rapid growth and development [2,7,8,9]. Maternal obesity influences the nutritional status of the child through different mechanisms, breastfeeding being one of them. If the mother of the child has obesity, the fatty acid (FA) profile in breast milk can be different, with a prevalence of pro-inflammatory FAs beyond those critical for neurodevelopment [10]. Thus, the early nutritional status and future health of the child can be affected.

Breast milk contains long-chain (LC) polyunsaturated fatty acids (PUFAs), which are crucial nutrients—especially docosahexaenoic (DHA) and arachidonic acid (AA)—involved in growth, the immune system, vision, and cognitive and motor development [11]. These nutrients are associated with the prevention of obesity [12,13] and other infectious and chronic diseases in the future life [14]. However, maternal characteristics, such as diet [15] or obesity [10], may alter the FA content in human milk. Studies have shown that the breast milk of mothers with overweight and obesity have higher levels of n6 FAs and lower levels of n3 FAs than the breast milk of normal-weight mothers [16,17,18], and a high ratio of n6:n3 LC-PUFAs in red blood cells membrane phospholipids has been reported as a risk factor for obesity [19]. In fact, in high-fat rodent models of maternal obesity, lowering the maternal n6:n3 ratio using a novel genetic model or supplemental fish oil has been shown to prevent offspring obesity [20]. Nevertheless, the results appear to be inconsistent [18,21].

The direct impact of maternal weight on the infant cognition has also been studied [21,22,23,24]. Mostly, observational, prospective and longitudinal studies correlate a high pre-pregnancy maternal body mass index (BMI) with poorer cognitive performance [24]. High gestational weight gain (GWG) seems to augment this correlation, as well [25]. However, three studies have failed to find an association between maternal obesity and cognitive infant deficits [26,27,28].

Although there are studies that have analyzed the influence of maternal weight on breast milk FA composition [10,18,29,30,31,32,33,34,35], none of these studies have further assessed its effect on infant cognition and growth. Furthermore, there is a lot of variability regarding the timing of breastmilk collection in the existing studies, and most of them focus on the analysis of mature breastmilk, without considering the evolution of the different FAs from colostrum to mature milk. Therefore, the current study aims to analyze the implications of maternal obesity on FA levels in colostrum and mature milk and their association with infant growth and cognition, to raise awareness about the programming effect of maternal nutrition and promote a healthy weight in women.

## 2. Materials and Methods

### 2.1. Statement of Ethics

This study was carried out in accordance with the ethical standards recognized by the Declaration of Helsinki (2004), the EEC Good Clinical Practice guidelines (document 111/3976/88 of July 1990) and current Spanish legislation governing clinical research in humans (Royal Decree 561/1993 on clinical trials). Additionally, the study was approved by San Cecilio University Hospital Ethics Committee and the Faculty of Medicine at the University of Granada. Written informed consent was obtained from all participants at the beginning of the study.

### 2.2. Study Population and Design

**Figure 1:**
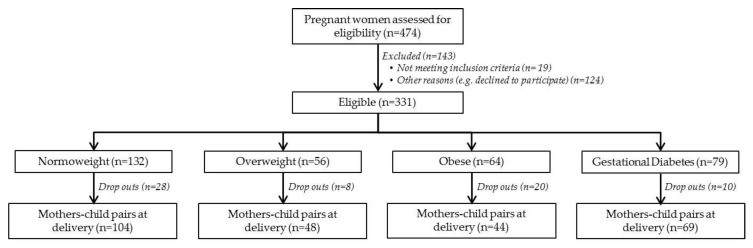
Participants in the PREOBE cohort and classification following BMI and gestational diabetes criteria.

For the present study, a subsample of mother–child pairs (*n* = 78) from the PREOBE cohort was selected and classified according to maternal pre-pregnancy BMI: normal-weight (BMI = 18.5–24.99 Kg/m^2^, *n* = 34), overweight (BMI = 25–29.99 Kg/m^2^, *n* = 27) and obese (BMI > 30 Kg/m^2^, *n* = 17). The PREOBE study (Role of Nutrition and Maternal Genetics on the Programming of Development of Fetal Adipose Tissue) is an observational cohort study of a total of 331 pregnant women that analyzes the impact of maternal obesity and gestational diabetes. The information regarding the PREOBE study has been published elsewhere [34] and was registered at www.ClinicalTrials.gov (NCT01634464). Figure 1 presents the study design and information of the PREOBE study.

Briefly, the study and recruitment of participants were carried out at San Cecilio University Hospital and the Mother-Infant Hospital in the city of Granada, Spain. The inclusion criteria were: singleton pregnancy, gestation between 12 and 20 weeks at enrollment, and an intention to deliver in one of the two obstetrics centers mentioned above. Women were excluded if they were participating in other research studies, receiving drug treatment or supplements of DHA or folate for more than the first three months of pregnancy, suffering from disorders such as hypertension, pre-eclampsia, fetal intrauterine growth retardation, infections, hypo- or hyperthyroidism and hepatic renal diseases, or following an unusual or vegan diet. Maternal age, pre-pregnancy BMI, parity, smoking status, diet, alcohol habits, socio-demographic information, education, gestational weight gain, infant anthropometry, gender and feeding practices were recorded. After birth, the women were encouraged to breastfeed their infants.

### 2.3. Breast Milk Sample Collection

Colostrum and mature milk were collected at 2–4 and 28–32 days postpartum, respectively, by an experienced nurse at the hospitals or by the mother at home (after receiving training by the nurse). Samples were collected over the course of an entire day (24 h) from both breasts before and after each feed. Milk samples were gathered in sterile polypropylene tubes by mechanically expressing each breast with a breast pump. Mothers were given 14 tubes with a capacity of 5 mL and the total volume obtained from each mother ranged from 45 to 70 mL. The samples collected at each time were frozen at −20 °C at home, and mothers brought them to the 3-month offspring follow-up visit. Each time, the samples were transported in ice boxes to the laboratory, where they were stored at −80 °C until analysis. All samples from each woman were mixed and aliquoted prior to analysis.

### 2.4. Fatty Acid Analysis of Breast Milk

The FA composition of breast milk was determined according to the method described by Chisaguano et al. [35]. 50 µL human milk samples were used for the analysis. FA methyl esters (FAMEs) were prepared with sodium methylate in methanol (0.5 M) and boron trifluoride methanol solution (14% v/v). They were then separated and quantified by fast gas chromatography (GC)using a HP-6890 Series GC System (Hewlett-Packard, Waldbronn, Germany) equipped with a flame ionization detector (FID), a split/splitless injector, a HP-7683B Series autoinjector, and a fused-silica SP-2560 capillary column (75 m 0.18 mm internal diameter, 0.14 µm thickness) coated with a 100% bis-cyanopropyl polysiloxane stationary phase (Supelco, Saunderton, UK). The chromatographic conditions used were: hydrogen as the carrier gas at a constant linear velocity of 22 cm/s (which gave an initial pressure of 39 psi). The detector and injector temperatures were set at 300 °C and 250 °C, respectively; the split ratio was at 1:50 and the injection volume was 1 µL. Oven temperatures were programmed as follows: the initial temperature was set at 120 °C, which was increased at a rate of 25 °C min^−1^ to 180 °C. This temperature was held for 6 min and finally increased to 240 °C at a rate of 25 °C min^−1^, and held for 9 min.

FAs were identified by a comparison of the peak retention times of those of the standard solution Supelco 37-component FAME mix (Sigma-Aldrich, St. Louis, MO, USA). FAs were then quantified by standard normalization (% total fatty acids), and they are therefore expressed as a percentage of the total amount of FAs. FA summatories were derived by adding the corresponding single FAs to saturated FAs (SFAs), monounsaturated FAs (MUFAs), PUFAs, n6 PUFAs, n3 PUFAs, n6 LC-PUFAs and n3 LC-PUFAs. Moreover, n6 to n3 ratios were created for analysis.

### 2.5. Assessment of Anthropometric Infant Outcomes

After birth, the infants received a medical examination during which anthropometric measurements were recorded. Data, such as weight, length and BMI at 6, 18 and 36 months of age were included in the present study. Length and weight (with light clothing and no shoes) were recorded using a Harpenden Infantometer (Model 702) calibrated stadiometer (Holtain, Wales, United Kingdom) and a Multina Comfort calibrated balance scale (SOEHNLE, Backnang, Germany), respectively. Weight, length and BMI measurements were ultimately converted to weight-for-age z-scores (WAZ), length-for-age z-scores (LAZ) and BMI-for-age z-scores (BMIZ) (SD scores), according to World Health Organization (WHO) child growth standards [36,37].

### 2.6. Assessment of Infant Cognitive Development

Infant cognitive development was assessed at 18 months of age using the Bayley Scales of Infant Development III (BSID III) [38], by trained psychologists in the presence of the mother of the child. These scales measure the level of motor, language and cognitive or mental development. The present study uses the Cognitive Composite score, which is the global score of the scales and represents the overall cognitive development of the children.

### 2.7. Statistical Analysis

Statistical analyses were performed using the SPSS statistical software package for Windows (version 23.0; SPSS Inc., Chicago, IL, USA). The Kolmogorov-Smirnov test was used to study the normal distribution of the data and non-normally distributed data were natural log-transformed. Means and standard deviations (SD) were used to describe continuous variables. The characteristics of the population were analyzed using the ANOVA and Bonferroni post-hoc test. To analyze the FA evolution from colostrum to mature milk, a paired Student’s *t*-test was used. The independent Student’s *t*-test was used to compare the breast milk FA composition between maternal weight groups. The associations between breast milk FAs and child anthropometric measurements and cognitive scores were determined using linear regression analyses and corrected for potential confounders such as maternal BMI, smoking, education, GWG and parity, and infant characteristics, such as gender and feeding practices. The Bonferroni correction (0.05/(48 FAs × 3 study groups = 144 analyses)) was applied to take multiple testing into account and *p*-value thresholds were set at 0.002. In the tables, *p*-values ≤ 0.05 are highlighted in bold, while those ≤0.002 are additionally marked by stars.

## 3. Results

### 3.1. Characteristics of the Population

The characteristics of the population are shown in Table 1. Normal-weight women presented the highest GWG, followed by overweight and finally mothers with obesity. The latter group had the highest n6:n3 ratio in dietary intake, while normal-weight mothers had the lowest intake of AA. No significant differences were found in infant characteristics according to maternal BMI.

### 3.2. Breast Milk Fatty Acid Evolution

The FA evolution from colostrum to mature milk is shown in Table 2. In spite of maternal pre-pregnancy BMI, mature breast milk presented lower levels of C16:1n9, C20:1n9, AA, C22:1n9, C22:4n6, C22:5n6, C22:5n3, DHA, C24:0, C24:1n6 and n3 LC-PUFAs, and higher levels of C8:0, C10:0, medium-chain FAs (MCFAs), eicosapentaenoic acid (EPA):AA and DHA:AA ratios than those found in colostrum.

Regarding other biologically important FAs, and always compared to colostrum levels, the mature milk of normal-weight mothers showed higher levels of C12:0 and C18:3n6, and lower levels of C16:0, C20:0, C20:3n6, C22:0, C22:2 n6, C23:0, saturated fatty acids (SFAs) and n3 PUFAs; the mature milk of overweight mothers showed increased levels of C6:0, C16:1n7, linoleic acid (LA), C18:3n6, EPA and n6:n3 ratio, and decreased concentrations of C15:0, C16:0, C17:0, C20:0, C23:0 and SFAs; and finally, the mature milk of mothers with obesity had higher levels of C6:0, C12:0 and C18:0 and lower levels of C18:1n7 and C22:0.

### 3.3. Breast Milk FAs According to Maternal Weight Group

Table 2 also shows the differences in breast milk FAs between weight groups. Compared to normal-weight women, the overweight group had higher levels of C14:1, C15:0, C17:0 and C17:1 in colostrum; and higher levels of C22:5n6 and n6:n3 ratio and lower levels of DHA and EPA:AA in mature milk.

On the other hand, compared to normal-weight mothers, mothers with obesity had lower levels of C18:0, C18:1n9t and ALA in colostrum; and lower levels of C18:1n9, C18:1n9t, ALA and MUFAs and higher levels of C22:2n6, C22:5n6, C23:0 and SFAs in mature milk.

We also compared overweight with mothers with obesity and found that the group with obesity had lower concentrations of C8:0, C15:0, C17:0, C18:1n9t, ALA in colostrum; and higher levels of C16:0 and SFAs and lower levels of ALA in mature milk.

### 3.4. Association of Breast Milk FAs with Infant Growth

Table 3 shows the associations between breast milk FAs and infant growth. All associations were observed after adjusting for potential confounders, which included maternal pre-pregnancy BMI, maternal smoking, weight gain during pregnancy, maternal education, gender of the child and type of infant feeding practice.

At 6 months of age, we found that colostrum levels of AA, EPA, DHA, n3 PUFAs, n6 LC-PUFAs and n3 LC-PUFAs were inversely associated with infant BMIZ, while the n6:n3 ratio was positively associated with it. Also, at 6 months of age, LA and the n6:n3 ratio in both colostrum and mature milk were positively associated with WAZ. No associations were found between mature milk and any variable at 1.5 or 3 years of age.

### 3.5. Associations of Breast Milk FAs with Infant Cognition

Table 4 presents the associations between breast milk PUFAs and infant cognition at 18 months of age. When the whole population was analyzed, no associations were found. However, infants born to normal-weight mothers presented a positive association between cognition scores and LA and n6 PUFA levels in colostrum. On the other hand, the infants of overweight mothers presented a direct association of DHA and n3 LC-PUFA levels in colostrum with cognitive score, while the n6:n3 ratio in colostrum was inversely associated with it. With respect to infants born to mothers with obesity, a positive association was found between ALA levels in mature milk and cognition.

## 4. Discussion

The present study offers the evaluation of human breast milk FA composition during the first month postpartum according to maternal weight, and the impact on child outcomes from 6 months to 3 years of age. This is one of the very few studies analyzing the influence of maternal weight on breast milk FA composition and, to our knowledge, the second one to assess this parameter in both colostrum and mature breast milk. Moreover, we believe this is the first study to address its effect on both infant cognitive developmental parameters and growth all in one study.

Upon analysis of the population characteristics, we observed that women with obesity had the lowest GWG, even though no nutritional intervention was carried out. This finding is in line with the results of a systematic review about GWG in women with obesity where they concluded that GWG decreased with each higher BMI classification [39]. In fact, weight loss during pregnancy is more common in women with obesity than non-obese women [40] and GWG decreases as the severity of obesity increases [41]. Nonetheless, we observed that women with obesity had the highest n6:n3 ratio in their dietary intake, which suggests they had the lowest-quality dietary intake, as similarly demonstrated by other studies where a high weight status is related to a high dietary intake of n6 FAs and a low intake of n3 FAs [42].

We did not observe any differences in infant characteristics according to maternal BMI. Evidence suggests that infants born to mothers with obesity have an increased risk of having a higher weight and length at birth [1], but this was not the case in our population. Since GWG is directly associated with birth weight [43], a possible explanation for this finding is that women with obesity showed the lowest GWG, and therefore their offspring did not present increased weight at birth. This is in the line with the conclusions of the systematic review of Faucher and Barger, in which several studies reported a linear decrease in the prevalence of being large for gestational age (LGA) and less GWG [39], suggesting that women with obesity and low GWG would have some benefits on fetal growth. This could be the reason why in our study children from women with obesity did not present higher weight or length. Nevertheless, our data still showed a tendency in which formula-fed infants and those considered LGA represented a higher percentage in the groups of women with overweight and obesity, according to Lubchenco’s curves [44].

Regardless of maternal weight, our data showed that when breast milk transitioned from colostrum (2–4 days postpartum) to mature milk (28–32 days postpartum), the levels of crucial FAs such as AA, DHA, and n3 LC-PUFA were decreased. Other authors have also demonstrated this finding [45,46]. The high content of crucial FAs in colostrum has biological relevance because it is highly associated with child outcomes, possibly because of the nutrient supply during the first few days of life, which are critical for infant health [47]. Nonetheless, we also found higher levels of EPA:AA and DHA:AA in mature milk, which are positively associated with health outcomes as well [48]. Analyzing the breast milk evolution within each weight group, we found that SFA concentrations decreased in the mature milk of women with normal-weight and overweight, but not in the mature milk of women with obesity. As described in other studies, the human milk of these women could present higher levels of SFA [17]. The factors attributed to this increased amount of SFA in breast milk of women with obesity could be the metabolic status and diet. It is well known that obesity is intrinsically a pro-inflammatory state influenced by dietary intake [49], where the ratio of n6:n3 PUFA is a clear factor affecting inflammation and obesity development [50]. In our study, the higher dietary intake of n6:n3 PUFA ratio in women with obesity, might be enhancing the pro-inflammatory state, and thereby affecting the levels of breast milk SFAs. Moreover, the fact that women with an increased BMI may have an increased intake of n6:n3 PUFA might also explain the higher n6:n3 PUFA ratio found in the mature milk of overweight women compared to their colostrum, although their increased intake of dietary n6:n3 was not significant.

To the best of our knowledge, eight studies have evaluated the FA composition of breast milk according to maternal BMI, but the results available in the literature are not entirely consistent and the studies differ in terms of the weight groups tested and the timing of sample collections. Out of these eight studies, only one shares the same collection timing for colostrum that we used [32]; another one used a similar timing for both colostrum and mature milk [16], but the other 6 studies collected the milk in different times ([10,17,18,29,30,31]). Regarding the weight groups used, 5 did not share the same groups that we used [10,16,17,29,31], and 2 out of the 3 studies that did [18,30,31] used a different criteria to classify weight according to BMI [30,32].

Although our results and the ones available in the literature suggest that a high maternal weight status alters human milk nutrient content, there is an inconsistency regarding which FAs are the most influenced according to BMI groups. This could be attributed to numerous factors, such as sample size, population, methods, FAs included in the analysis, weight group classification and the timing of breast milk collection. However, it is important to highlight that, even without a clear consistency among studies, an increased BMI is found to alter FA concentrations in breast milk, generally increasing SFA and n6 PUFAs and decreasing FAs from the n3 series. An important factor that could explain the differences found among weight groups could be related to dietary intake during late pregnancy, since several studies have demonstrated that this affects breast milk composition [51]. This suggests that women with overweight and obesity could have an increased dietary intake of n6 FAs and SFAs and a poor intake of n3 FAs. As previously mentioned, this happened in our population, where we found that the n6:n3 ratio of dietary intake was higher in women with an increased BMI, especially those with obesity. Since a maternal pro-inflammatory diet is positively associated with increased concentrations of SFA and MUFA in breast milk [10], specific maternal metabolic markers could be an interesting approach to predicting the predominance of certain FAs in breast milk.

This study also analyzed the possible association between breast milk FA composition and infant growth and cognition. It is well known that many nutrients are critical for proper infant growth and neurodevelopment. Animal models and epidemiological studies suggest that PUFAs such as AA and DHA are particularly important [52,53]. Thus, we evaluated the association between the PUFA levels in breast milk and infant anthropometric measurements at 6, 18 and 36 months of age. For this analysis, we used the z-score values WAZ, LAZ and BMIZ to evaluate with greater accuracy which children were within or outside the normal range [1,36,54]. Our findings showed that LC-PUFAs—especially AA, EPA, DHA, n3 and n6 LC-PUFAs—in colostrum had a negative association with infant BMIZ at 6 months. In accordance with these results, a recent review that analyzed the association between n3 PUFAs and growth suggested that DHA during pregnancy, lactation and early life may be associated with significant benefits in infant growth and development [55]. Similarly, Pedersen et al. observed a negative association between DHA levels in breast milk and BMI in children from 2 to 7 years of age. They also found an overall inverse association between breast milk DHA and body fat percentage [56]. Although it is important to mention that BMI is not the best method to quantify body composition, and especially to assess body fat in children [57,58], DHA content in breast milk could have some benefits in postponing the age of adiposity rebound [56], which is the second rise in adiposity that usually occurs between 3 and 7 years of age [59]. It is known that the age that rebound occurs predicts later fatness, meaning that an earlier rebound would be a risk factor for later obesity [59]. On the other hand, our data also indicated that n6 PUFA levels may contribute to a fat mass increase in children [59], since LA in colostrum and the n6:n3 ratio in both mature milk and colostrum could influence WAZ and BMIZ at 6 months of age. Since the n6 PUFAs in mature milk were generally increased in overweight and obese mothers, their children could be more susceptible to developing obesity [17,20,50]. Indeed, children from overweight and obese mothers presented a tendency to be LGA. In contrast, Much et al. found that AA and n6 PUFAs in mature breast milk were negatively associated with infant weight and BMI (up to 4 months of age) [33], suggesting that the role of these n6 FAs (including AA) might be age-dependent and serve as important regulating factors for growth in early postnatal life. Due to the low variability of AA contents in breast milk across populations (0.24–1% of FAs) [7], a possible explanation of this discrepancy could be the quantitative amount of milk intake by the breastfed infant, meaning that depending on the daily ingested volume of milk, AA would have its growth-regulatory effects or not [33]. Further studies are needed to look into such quantitative aspects. In our study, we only found significant associations between FAs and infant growth at 6 months of life, but not at 18 nor 36. This finding may be due to the child’s own diet, lifestyle and metabolism. However, the associations found at 6 months are relevant, because it is a crucial age that represents a critical period in the child’s development and programming [3]. A curious result that we found is that length was not correlated to any PUFA, which again is in disagreement with Much et al. They inversely correlated DHA, EPA and n3 PUFA with length at 1 year of age. Their milk collection was at 6 weeks and 4 months postpartum [33], whereas in our study it was at 2–4 days and 28–32 days postpartum. Therefore, the possible evolution of FA species over time would be a possible explanation for the different results. From our study and the evidence gathered, we can see that PUFAs in breastmilk influence infant growth; however, there is a high variability in existing results. Further studies are needed to obtain more conclusive outcomes [2].

PUFAs are also critical for an adequate brain growth and function in aspects such as neurogenesis, nerve impulse transmission, neuronal integrity, and vitality and gene expression in the brain [52,53,60]. Thus, we explored the association between breast milk PUFA levels and cognitive score at 18 months of life. On the one hand, when we analyzed the total population, we found no association between any FA in breast milk and child development. Similarly, there have been observational studies that found no strong evidence for a beneficial role of LC-PUFAs in order to explain the positive relationship between breastfeeding and cognition [61]. This raises the question as to whether LC-PUFA levels may only be beneficial in children’s mental development when breastfeeding levels are high [62]. Although we corrected the analysis by the type of breastfeeding, this information was collected at 3 months of age, so we do not know which effect could have had a longer period of exclusive breastfeeding.

We also explored the association between breast milk FA levels and infant cognition according to maternal BMI. In general, we found a direct association between n3 and n6 PUFA levels in colostrum and infant cognition at 18 months of age. The colostrum from overweight mothers was the one that presented more relevant associations, specifically, a high n6:n3 ratio was negatively associated with cognition, whereas higher DHA concentrations were directly associated with better cognitive scores, which is in line with Bernard et al. [63]. This suggests that the cognition of infants born to overweight women could be enhanced by promoting n3 FAs, more specifically DHA, in the maternal diet. These results are in line with meta-analyses, animal and epidemiologic studies [60,64,65], and support WHO recommendations on breastfeeding for the two first years of life or beyond [66]. We must consider that, in our study, the cognitive score was assessed at 1.5 years of life, and at this age, there are many factors related to the child that could influence their cognition. The potential cofounders that we have used to adjust this analysis were mainly related to the mother, and only the gender and type of feeding practice were related to the child. Important factors such as infant diet or physical activity are lacking and could have a huge influence in the results because intake of micronutrients, such as n3 FAs, vitamin B12, folic acid, zinc, iron and iodine, together with malnutrition and general dietary patterns and other lifestyle habits, influence child cognitive development as well [67,68,69].

Overall, our study highlights the importance of the maternal health before, during and after pregnancy, since it could have a great impact in the breast milk FA composition and, in consequence, in the offspring’s growth and cognition which affects their future health. Many women start developing healthy habits when they are pregnant or planning a pregnancy. However, as presented in our study, the pre-pregnancy health status has an important effect in the quality of the human milk, consequently affecting the health of the child. Therefore, bigger efforts must be put in place to promote and guarantee a healthier lifestyle and nutritional status in the general population to pursuit healthier future generations.

We acknowledge some limitations in our study, such as the small sample size. However, it is important to understand that, even though PREOBE is a larger cohort, we were not able to include all the participants in the present study due to lack of data or samples, possibly related to indisposition to participate given the complexity and sensitivity of the periods involved: childbirth and breastfeeding. Although risk factors, such as socio-demographic information and maternal diet, allowed us to adjust our statistical models for potential confounders, we cannot rule out residual confounding, especially coming from data related to the infants at 1.5 and 3 years of age because data on their dietary intake, lifestyle and other characteristics, could be greatly influencing the results. Another limitation is that women receiving supplements of DHA for over 3 months were excluded, but we do not know the possible effect of that initial supplementation in the breast milk FA profile. Moreover, recording the timing between sample collection or the last meal, collecting information on what was consumed before and after each sample was taken, and analyzing the different breast milk samples of one day without mixing them, would provide valuable data to assess the human milk nutrient content and impact. In general, further research is required to provide a better understanding of the role that FAs play in obesity development and management, paying special attention to the methods used for analysis and promoting the comparison of results between cohorts.

## 5. Conclusions

In conclusion, our results show that (1) the FA composition of colostrum and mature milk was different. Regardless of maternal weight, mature milk had lower levels of AA and DHA (among others) than colostrum; (2) Maternal obesity influenced the FA concentrations in breast milk. Overall, breast milk of mothers with a high BMI presented increased SFA levels and n6:n3 ratio, and decreased ALA, DHA and MUFA concentrations; and (3) The early supply of n6 and n3 PUFAs through colostrum influenced infant weight status and cognition, at 6 and 18 months of life, respectively. Infant BMIZ at 6 months of age was inversely associated with colostrum levels of n6 and n3 LC-PUFAs (e.g., AA and DHA) and positively associated with n6:n3 ratio. Depending on the maternal BMI, infant cognition may be positively affected by colostrum levels of LA, n6 PUFAs, DHA, n3 LC-PUFAs and ALA, and negatively affected by the n6:n3 ratio. Since the maternal pre-pregnancy weight can influence the breast milk FAs, which is related to the early nutritional status of the child and to health conditions throughout the life span, this study endorses the need for early preventive health care through diet and lifestyle. A healthy weight in women before, during and after pregnancy should be encouraged to promote beneficial FAs in breast milk and promote healthier future generations.

## Figures and Tables

**Table 1:** Characteristics of the population.

Characteristic		Normal-Weight		Overweight		Obesity	*p*
		Mean (SD)		Mean (SD)		Mean (SD)	
Maternal characteristics	*n*		*n*		*n*		
Age (years)	34	31 (4)	27	32 (4)	17	32 (4)	0.492
Pre-pregnancy BMI (kg/m^2^)	34	22.14 (1.54) ^a^	27	27.59 (1.35) ^b^	17	33.40 (2.65) ^c^	<0.001 *
Weight Gain (kg)	25	13.17 (3.55)	23	10.32 (5.20)	15	9.14 (7.06)	**0.042**
Education (%)							0.660
<High school	26	14.71	19	11.11	11	23.53	
High school	3	8.82	5	18.52	2	11.76	
>High school	5	76.47	3	70.37	4	64.71	
Smoking during pregnancy (%)							0.415
No, never	17	77.27	15	71.43	10	73.68	
Yes	3	13.64	5	23.81	1	7.14	
Quit	2	9.09	1	4.76	3	21.43	
Maternal dietary intake							
Energy (Kcal/day)	27	2066.37 (261.93)	23	2089.59 (542.07)	12	2058.08 (469.97)	0.961
Lipids (g)	27	86.89 (17.29)	23	85.25 (25.86)	12	93.23 (20.95)	0.468
Lipids (%)	27	37.76 (5.26)	23	39.04 (7.73)	12	41.17 (5.38)	0.307
SFA(g/d)	27	30.81 (6.36)	23	30.05 (8.67)	12	34.19 (5.87)	0.203
MUFA (g/d)	27	36.53 (10.92)	23	39.33 (17.63)	12	36.32 (12.08)	0.926
PUFA(g/d)	27	12.10 (3.17)	23	13.34 (6.59)	12	14.63 (3.95)	0.285
n6 PUFA (g/d)	27	2.48 (1.95)	23	2.89 (2.53)	12	3.44 (1.50)	0.122
n3 PUFA (g/d)	27	0.18 (0.11)	23	0.21 (0.13)	12	0.20 (0.09)	0.427
*n*-3 from fish (g/d)	27	0.36 (0.31)	23	0.28 (0.34)	12	0.45 (0.34)	0.690
AA (g/d)	27	0.11 (0.06) ^a^	23	0.17 (0.08) ^b^	12	0.16 (0.08) ^ab^	**0.005**
EPA(g/d)	27	0.12 (0.11)	23	0.09 (0.11)	12	0.16 (0.12)	0.213
DHA (g/d)	27	0.24 (0.18)	23	0.22 (0.21)	12	0.31 (0.21)	0.269
n6:n3	27	12.99 (2.98) ^a^	23	13.53 (3.40) ^a^	12	19.12 (8.94) ^b^	**0.004**
Infant characteristics							
Sex, male (%)	14	41.18	11	40.74	7	41.18	0.999
Birth weight (g)	32	3359.06 (352.35)	27	3340.37 (511.85)	16	3532.35 (389.61)	0.277
Birth length (cm)	31	50.52 (1.57)	27	50.30 (1.88)	16	51.22 (1.80)	0.245
Birth head Circumference (cm)	26	34.31 (1.36)	22	34.36 (1.39)	16	34.69 (1.40)	0.674
Placenta (g)	30	496.67 (144.11)	25	509.20 (130.25)	16	568.13 (143.17)	0.332
Newborn according Lubchenco curves ^#^ (%)							0.627
SGA	0	0.00	1	4.55	0	0.00	
AGA	26	81.25	16	72.73	11	73.33	
LGA	6	18.75	5	22.73	4	26.67	
Breastfeeding ^†^ (%)							0.290
Exclusive	16	53.33	16	66.67	8	50.00	
Mixt	10	33.33	3	12.50	3	18.75	
Artificial	4	13.33	5	20.83	5	31.25	

Different superscript letters indicate differences among BMI groups, according to ANOVA and the Bonferroni post-hoc test. Chi-square test was applied to qualitative variables. *p*-values ≤ 0.05 are highlighted in bold and those ≤0.002 are additionally marked by stars. ^#^ The newborns were divided into three groups according to the Lubchenco curves: SGA: Small for Gestational Age; AGA: Appropriate for Gestational Age; LGA: Large for Gestational Age (LGA). ^†^ Breastfeeding practice information was collected at 3 months of age of the child. SFA: Saturated Fatty Acids; MUFA: Monounsaturated Fatty Acids; PUFA: Polyunsaturated Fatty Acids; AA: Arachidonic Acid; EPA: Eicosapentaenoic Acid; DHA: Docosahexaenoic Acid.

**Table 2:** Human milk fatty acids profile according to maternal pre-pregnancy BMI.

	Normal-Weight			Overweight			Obesity		
	Colostrum (*n* = 26)	Mature Milk (*n* = 20)	*p*	Colostrum (*n* = 21)	Mature Milk (*n* = 23)	*p*	Colostrum (*n* = 16)	Mature Milk (*n* = 14)	*p*
	Mean (SD)	Mean (SD)		Mean (SD)	Mean (SD)		Mean (SD)	Mean (SD)	
C6:0	0.06 (0.04)	0.08 (0.03)	0.19	0.05 (0.03)	0.10 (0.04)	<0.001 *	0.05 (0.03)	0.08 (0.07)	0.002 *
C8:0	0.13 (0.09)	0.23 (0.07)	0.002 *	0.13 (0.08)	0.24 (0.09)	<0.001 *	**0.08 (0.03) ^#^**	0.24 (0.10)	<0.001 *
C10:0	0.86 (0.57)	1.29 (0.37)	**0.010**	0.86 (0.47)	1.36 (0.33)	0.002 *	0.70 (0.57)	1.47 (0.32)	<0.001 *
C12:0	4.10 (2.07)	4.86 (1.80)	**0.039**	4.18 (1.97)	4.86 (1.92)	0.14	3.67 (2.49)	5.32 (1.65)	**0.029**
C14:0	5.15 (1.41)	4.79 (1.71)	0.88	5.38 (1.92)	4.67 (1.66)	0.38	5.04 (2.31)	5.23 (1.22)	0.32
C14:1	0.08 (0.02)	0.10 (0.03)	0.20	**0.11 (0.03) ^†^**	0.11 (0.04)	0.51	0.10 (0.05)	0.12 (0.06)	0.10
C15:0	0.21 (0.03)	0.21 (0.06)	0.80	**0.24 (0.04) ^†^**	0.20 (0.05)	**0.016**	**0.19 (0.04) ^#^**	0.22 (0.08)	0.32
C16:0	21.13 (2.32)	19.56 (2.29)	**0.006**	21.24 (1.46)	19.53 (2.02)	0.001 *	21.96 (2.11)	**21.20 (2.55) ^#^**	0.42
C16:1*t*	0.12 (0.03)	0.13 (0.04)	0.59	0.14 (0.04)	0.11 (0.05)	0.06	0.13 (0.04)	0.13 (0.06)	0.98
C16:1n9	0.50 (0.05)	0.43 (0.06)	<0.001 *	0.54 (0.13)	0.45 (0.07)	**0.010**	0.54 (0.10)	0.46 (0.10)	**0.024**
C16:1n7	1.48 (0.45)	1.83 (0.62)	0.11	1.63 (0.36)	1.80 (0.49)	**0.014**	1.78 (0.60)	1.88 (0.58)	0.61
C17:0	0.30 (0.04)	0.29 (0.08)	0.74	**0.34 (0.03) ^†^**	0.29 (0.06)	**0.026**	**0.29 (0.05) ^#^**	0.32 (0.07)	0.28
C17:1	0.15 (0.03)	0.17 (0.03)	0.28	**0.18 (0.04) ^†^**	0.18 (0.04)	0.88	0.17 (0.07)	0.17 (0.05)	0.83
C18:0	5.74 (0.67)	5.74 (0.41)	0.20	5.58 (0.85)	5.88 (0.67)	0.12	**5.44 (0.81) ^†^**	6.03 (0.49)	<0.001 *
C18:1n9	38.47 (3.51)	39.69 (4.23)	0.41	38.16 (4.57)	38.73 (5.53)	0.87	37.36 (3.11)	**36.63 (0.96) ^†^**	0.30
C18:1n9*t*	0.33 (0.14)	0.28 (0.09)	0.12	0.31 (0.09)	0.26 (0.08)	0.24	**0.24 (0.05) ^†#^**	**0.22 (0.04) ^†^**	0.15
C18:1n7	1.69 (0.22)	1.58 (0.24)	0.02	1.69 (0.28)	1.63 (0.23)	0.81	1.89 (0.42)	1.59 (0.32)	**0.022**
C18:1n-7*t*11	0.28 (0.12)	0.30 (0.12)	0.95	0.28 (0.09)	0.26 (0.11)	0.15	0.27 (0.14)	0.24 (0.08)	0.72
C18:2n6 (LA)	13.03 (2.42)	13.60 (3.21)	0.31	13.31 (3.20)	15.20 (3.86)	**0.050**	12.98 (2.74)	13.90 (3.14)	0.49
C18:2n7*c*9*t*11	0.12 (0.03)	0.13 (0.05)	0.69	0.14 (0.04)	0.12 (0.04)	0.32	0.13 (0.05)	0.14 (0.06)	0.67
C18:3n6	0.09 (0.04)	0.17 (0.06)	**0.010**	0.11 (0.07)	0.18 (0.05)	<0.001 *	0.13 (0.09)	0.17 (0.05)	0.15
C18:3n3 (ALA)	0.54 (0.18)	0.59 (0.21)	0.61	0.53 (0.13)	0.58 (0.16)	0.14	**0.41 (0.04) ^†#^**	**0.46 (0.08) ^†#^**	0.16
C20:0	0.20 (0.03)	0.18 (0.01)	**0.012**	0.18 (0.02)	0.17 (0.03)	**0.005**	0.19 (0.04)	0.18 (0.04)	0.75
C20:1n9	0.77 (0.20)	0.50 (0.07)	<0.001 *	0.74 (0.26)	0.46 (0.05)	<0.001 *	0.77 (0.24)	0.48 (0.06)	<0.001 *
C20:3n6	0.60 (0.15)	0.47 (0.11)	<0.001 *	0.65 (0.21)	0.48 (0.06)	**0.004**	0.68 (0.24)	0.52 (0.14)	0.05
C20:4n6 (AA)	0.67 (0.19)	0.49 (0.05)	**0.005**	0.66 (0.13)	0.49 (0.12)	<0.001 *	0.67 (0.23)	0.47 (0.10)	**0.004**
C20:5n3 (EPA)	0.05 (0.02)	0.07 (0.03)	0.07	0.04 (0.02)	0.06 (0.02)	<0.001 *	0.05 (0.03)	0.07 (0.02)	0.10
C22:0	0.08 (0.03)	0.06 (0.02)	**0.007**	0.09 (0.03)	0.08 (0.03)	0.20	0.08 (0.02)	0.07 (0.02)	**0.039**
C22:1n9	0.18 (0.06)	0.09 (0.02)	<0.001 *	0.19 (0.08)	0.09 (0.02)	<0.001 *	0.18 (0.06)	0.09 (0.01)	<0.001 *
C22:2n6	0.07 (0.03)	0.04 (0.01)	**0.003**	0.08 (0.03)	0.06 (0.03)	0.22	0.06 (0.02)	**0.06 (0.02) ^†^**	0.22
C22:4n6	0.25 (0.13)	0.10 (0.02)	<0.001 *	0.28 (0.15)	0.12 (0.04)	<0.001 *	0.37 (0.27)	0.12 (0.03)	<0.001 *
C22:5n6	0.12 (0.04)	0.05 (0.01)	<0.001 *	0.12 (0.05)	**0.07 (0.03) ^†^**	**0.048**	0.12 (0.05)	**0.09 (0.05) ^†^**	**0.040**
C22:5n3	0.16 (0.08)	0.11 (0.03)	**0.012**	0.14 (0.05)	0.11 (0.03)	0.002 *	0.19 (0.09)	0.12 (0.04)	**0.007**
C22:6n3 (DHA)	0.41 (0.14)	0.28 (0.11)	<0.001 *	0.35 (0.08)	**0.22 (0.06 ^†^**	<0.001 *	0.39 (0.13)	0.25 (0.07)	0.002 *
C23:0	0.12 (0.05)	0.05 (0.02)	<0.001 *	0.12 (0.05)	0.06 (0.03)	<0.001 *	0.13 (0.05)	0.09 (0.07) ^†^	0.06
C24:0	0.10 (0.03)	0.05 (0.02)	0.002 *	0.10 (0.04)	0.06 (0.03)	**0.007**	0.09 (0.03)	0.06 (0.03)	**0.031**
C24:1	0.20 (0.11)	0.06 (0.02)	<0.001 *	0.19 (0.13)	0.06 (0.04)	<0.001 *	0.19 (0.09)	0.07 (0.03)	<0.001 *
EPA:AA	0.07 (0.03)	0.12 (0.03)	**0.005**	0.06 (0.03)	**0.09 (0.04) ^†^**	<0.001 *	0.07 (0.03)	0.12 (0.07)	**0.004**
DHA:AA	0.63 (0.22)	0.77 (0.19)	**0.005**	0.55 (0.13)	0.76 (0.24)	<0.001 *	0.61 (0.21)	0.90 (0.34)	**0.004**
SFA	27.45 (2.66)	25.83 (2.48)	**0.008**	27.43 (1.93)	25.95 (2.49)	**0.004**	27.96 (2.34)	**27.80 (2.62) ^†,#^**	0.96
MCFA	5.14 (2.69)	6.46 (2.19)	**0.021**	5.23 (2.46)	6.56 (2.27)	**0.037**	4.50 (3.06)	7.11 (1.99)	**0.010**
MUFA	42.80 (3.51)	43.73 (4.17)	0.87	42.61 (4.91)	42.76 (5.70)	0.74	42.17 (3.56)	**40.74 (1.37) ^†^**	0.12
n6 PUFA	15.41 (2.53)	15.24 (3.31)	0.77	15.79 (3.23)	16.89 (3.93)	0.29	15.67 (3.01)	15.64 (3.19)	0.93
n3 PUFA	1.16 (0.30)	1.05 (0.24)	**0.018**	1.07 (0.15)	0.96 (0.21)	0.12	1.04 (0.23)	0.90 (0.13)	0.06
n6 LC-PUFA	2.30 (0.59)	1.48 (0.22)	<0.001 *	2.36 (0.63)	1.52 (0.24)	<0.001 *	2.56 (0.93)	1.58 (0.34)	<0.001 *
n3 LC-PUFA	0.62 (0.22)	0.45 (0.16)	<0.001 *	0.54 (0.12)	0.38 (0.11)	<0.001 *	0.63 (0.23)	0.44 (0.09)	**0.008**
n6:n3 PUFA	14.28 (4.76)	15.08 (3.91)	0.06	15.13 (4.37)	**18.28 (5.33) ^†^**	**0.033**	15.67 (4.22)	17.80 (4.97)	0.15
n6:n3 LC-PUFA	4.00 (1.16)	3.62 (1.18)	0.08	4.49 (0.95)	4.28 (1.24)	0.41	4.23 (1.05)	3.73 (0.92)	0.14

Means are expressed as percentages of total FAs. The *p*-value shown refers to the breast milk evolution within each group of weight, according to Student’s paired *t*-test. *p*-values ≤ 0.05 are highlighted in bold and those ≤0.002 are additionally marked by stars. ^†^ Indicates differences (*p* ≤ 0.05) compared with normal-weight group and its corresponding breast milk, according to Student’s independent *t*-test. ^#^ Indicates differences (*p* ≤ 0.05) between overweight and obese groups and corresponding breast milk, according to Student’s independent *t*-test; LA: Linoleic Acid; AA: Arachidonic Acid; ALA: α-linolenic Acid; EPA: Eicosapentaenoic Acid; DHA: Docosahexaenoic Acid; SFA: Saturated Fatty Acids; MCFA: Medium-chain Fatty Acids; MUFA: Monounsaturated Fatty Acids; PUFA: Polyunsaturated Fatty Acids; LC-PUFA: Long-Chain Polyunsaturated Fatty Acids.

**Table 3:** Associations between breast milk PUFA levels and anthropometric measurements in infants.

Fatty Acid	BMIZ				WAZ				LAZ			
	Colostrum		Mature Milk		Colostrum		Mature Milk		Colostrum		Mature Milk	
	6mo *n* = 37		6mo *n* = 39		6mo *n* = 37		6mo *n* = 39		6mo *n* = 38		6mo *n* = 39	
	18mo *n* = 38		18mo *n* = 37		18mo *n* = 38		18mo *n* = 38		18mo *n* = 38		18mo *n* = 38	
	36mo *n* = 16		36mo *n* = 13		36mo *n* = 16		36mo *n* = 13		36mo *n* = 18		36mo *n* = 14	
	β	*p*	β	*p*	β	*p*	β	*p*	β	*p*	β	*p*
C18:3n3 (ALA)												
6mo	−0.11	0.63	−0.15	0.40	−0.06	0.77	−0.22	0.24	0.05	0.80	−0.12	0.49
18mo	0.32	0.13	−0.11	0.57	0.11	0.63	−0.13	0.51	−0.20	0.26	−0.06	0.69
36mo	−0.44	0.21	0.01	0.99	−0.27	0.52	0.06	0.95	−0.17	0.63	−0.86	0.22
C18:2n6 (LA)												
6mo	0.33	0.12	−0.13	0.43	**0.42**	**0.027**	0.16	0.36	0.15	0.40	0.09	0.60
18mo	−0.19	0.34	0.04	0.85	0.18	0.37	0.05	0.79	0.06	0.73	0.17	0.26
36mo	0.12	0.75	−0.22	0.50	0.02	0.96	−0.136	0.75	0.01	0.98	−0.33	0.41
C20:4n6 (AA)												
6mo	**−0.44**	**0.016**	0.02	0.91	−0.20	0.26	0.04	0.84	0.25	0.13	0.03	0.86
18mo	−0.03	0.89	−0.12	0.57	0.06	0.77	−0.13	0.52	0.10	0.50	0.15	0.39
36mo	0.30	0.30	0.09	0.81	0.42	0.20	0.07	0.86	0.20	0.48	0.12	0.75
C20:5n3 (EPA)												
6mo	**−0.51**	**0.012**	0.00	0.99	−0.36	0.07	−0.13	0.49	0.18	0.31	−0.18	0.29
18mo	−0.30	0.12	0.00	0.98	−0.13	0.51	0.08	0.66	0.15	0.35	0.09	0.62
36mo	−0.74	0.155	−1.08	0.30	−0.58	0.34	−1.13	0.28	0.14	0.69	−0.59	0.61
C22:6n3 (DHA)												
6mo	**−0.37**	**0.043**	−0.16	0.38	−0.31	0.07	−0.29	0.10	0.00	0.99	−0.23	0.17
18mo	0.14	0.42	0.03	0.88	0.08	0.66	0.00	0.99	−0.05	0.74	−0.03	0.84
36mo	0.42	0.29	0.33	0.46	0.65	0.13	0.38	0.39	0.46	0.22	0.58	0.14
n6 PUFA												
6mo	0.21	0.32	0.13	0.45	0.34	0.07	0.16	0.35	0.20	0.27	0.10	0.55
18mo	0.16	0.41	−0.06	0.776	0.18	0.40	0.04	0.83	0.08	0.65	0.18	0.23
36mo	0.20	0.587	−0.19	0.64	0.15	0.72	−0.11	0.79	0.07	0.84	−0.31	0.44
n3 PUFA												
6mo	**−0.38**	**0.047**	−0.19	0.27	−0.33	0.07	−0.32	0.07	−0.00	0.991	−0.22	0.18
18mo	0.16	0.38	−0.11	0.56	0.04	0.84	−0.11	0.53	−0.12	0.427	−0.05	0.75
36mo	−0.20	0.60	0.17	0.78	0.05	0.90	0.21	0.74	0.05	0.897	−0.18	0.77
n6 LC−PUFA												
6mo	**−0.38**	**0.047**	−0.06	0.77	−0.17	0.36	0.00	0.98	0.19	0.253	0.09	0.65
18mo	−0.05	0.77	−0.27	0.19	0.03	0.88	−0.17	0.41	0.10	0.508	0.19	0.25
36mo	0.40	0.22	0.11	0.78	0.60	0.09	0.12	0.76	0.25	0.390	−0.03	0.95
n3 LC−PUFA												
6mo	**−0.43**	**0.020**	−0.19	0.28	−0.34	0.05	−0.33	0.06	0.03	0.866	−0.24	0.16
18mo	0.07	0.70	−0.04	0.82	0.05	0.78	−0.02	0.90	−0.01	0.955	0.02	0.89
36mo	0.28	0.44	0.19	0.63	0.53	0.18	0.21	0.59	0.42	0.211	0.40	0.31
n6:n3												
6mo	**0.42**	**0.031**	0.30	0.10	**0.45**	**0.011**	**0.45**	**0.013**	0.11	0.519	0.30	0.08
18mo	−0.04	0.82	0.05	0.78	0.06	0.74	0.14	0.47	0.14	0.369	0.21	0.19
36mo	0.30	0.34	−0.23	0.55	0.05	0.88	−0.18	0.65	0.01	0.978	−0.26	0.58
LC n6:n3												
6mo	0.12	0.56	0.14	0.41	0.22	0.24	0.29	0.09	0.15	0.373	0.24	0.13
18mo	−0.13	0.48	−0.08	0.66	−0.04	0.86	−0.05	0.77	0.11	0.490	0.07	0.65
36mo	0.35	0.54	−0.07	0.86	0.16	0.81	−0.08	0.85	−0.31	0.516	−0.30	0.46

Associations were evaluated using lineal regression analyses. β and *p* are corrected values after adjustment for potential confounders: maternal pre-pregnancy BMI, maternal smoking, weight gain during pregnancy, maternal education, sex of the child and type of infant feeding practice. *p*-values ≤ 0.05 are highlighted in bold and those ≤0.002 are additionally marked by stars. mo: month; LA: Linoleic Acid; AA: Arachidonic Acid; ALA: α-linolenic Acid; EPA: Eicosapentaenoic Acid; DHA: Docosahexaenoic Acid; PUFA: Polyunsaturated Fatty Acids; LC-PUFA: Long chain Polyunsaturated Fatty Acids.

**Table 4:** Associations between breast milk PUFA levels and infant cognition score at 18 months of age, according to maternal pre-pregnancy BMI.

Fatty Acid	All				Normal-Weight				Overweight				Obesity			
	Colostrum (*n* = 75)		Mature Milk (*n* = 77)		Colostrum (*n* = 14)		Mature Milk (*n* = 12)		Colostrum (*n* = 11)		Mature Milk (*n* = 15)		Colostrum (*n* = 12)		Mature Milk (*n* = 11)	
	β	*p*	β	*p*	β	*p*	β	*p*	β	*p*	β	*p*	β	*p*	β	*p*
C18:3n3 (ALA)	0.08	0.718	0.01	0.942	0.29	0.581	0.82	0.212	0.44	0.393	−0.15	0.662	0.55	0.468	**2.34**	**0.008**
C18:2n6 (LA)	0.20	0.339	0.12	0.527	**0.84**	<0.001 *	0.88	0.069	−0.95	0.061	−0.16	0.687	0.12	0.869	0.53	0.470
C20:4n6 (AA)	0.03	0.889	−0.20	0.333	−1.23	0.136	−0.56	0.270	0.32	0.594	−0.28	0.544	0.48	0.416	−0.87	0.405
C20:5n3 (EPA)	0.31	0.133	−0.11	0.580	0.00	0.996	0.59	0.428	−0.07	0.932	−0.18	0.635	0.80	0.233	−0.29	0.658
C22:6n3 (DHA)	−0.16	0.396	−0.27	0.161	−0.73	0.124	−0.19	0.720	**0.88**	**0.045**	0.33	0.362	−0.03	0.954	−1.16	0.104
n6 PUFA	0.24	0.251	0.13	0.495	**0.81**	0.002 *	0.88	0.064	−0.97	0.111	−0.15	0.713	0.16	0.803	0.45	0.520
n3 PUFA	−0.01	0.966	−0.12	0.541	−0.23	0.687	0.37	0.512	0.70	0.057	−0.04	0.922	0.13	0.829	−0.78	0.489
n6 LC-PUFA	0.14	0.446	0.18	0.407	0.01	0.984	0.31	0.536	0.53	0.227	0.36	0.481	0.28	0.650	−0.09	0.924
n3 LC-PUFA	−0.11	0.563	−0.25	0.190	−0.71	0.113	−0.08	0.873	**1.01**	**0.004**	0.21	0.585	0.04	0.949	−0.89	0.189
n6:n3	0.13	0.489	0.22	0.255	0.74	0.067	0.29	0.639	**−0.97**	0.002 *	−0.10	0.805	0.01	0.985	0.57	0.426
LC n6:n3	0.27	0.166	0.29	0.105	0.52	0.172	0.16	0.752	0.05	0.952	−0.08	0.830	0.22	0.696	0.83	0.286

Associations were evaluated using lineal regression analyses. β and *p* are corrected values after adjustment for potential confounders: maternal smoking, weight gain during pregnancy, maternal education, sex of the child and type of infant feeding practice. Cognition performance was analyzed at 1.5 years of age. *p*-values ≤ 0.05 are highlighted in bold and those ≤0.002 are additionally marked by stars. LA: Linoleic Acid; AA: Arachidonic Acid; ALA: α-linolenic Acid; EPA: Eicosapentaenoic Acid; DHA: Docosahexaenoic Acid; PUFA: Polyunsaturated Fatty Acids; LC-PUFA: Long chain Polyunsaturated Fatty Acids.
